# The role of microglia and myeloid immune cells in acute cerebral ischemia

**DOI:** 10.3389/fncel.2014.00461

**Published:** 2015-01-14

**Authors:** Corinne Benakis, Lidia Garcia-Bonilla, Costantino Iadecola, Josef Anrather

**Affiliations:** Feil Family Brain and Mind Research Institute, Weill Cornell Medical CollegeNew York, NY, USA

**Keywords:** cerebral ischemia, monocytes, microglia, neutrophils, myeloid cells, tissue macrophages

## Abstract

The immune response to acute cerebral ischemia is a major contributor to stroke pathobiology. The inflammatory response is characterized by the participation of brain resident cells and peripheral leukocytes. Microglia in the brain and monocytes/neutrophils in the periphery have a prominent role in initiating, sustaining and resolving post-ischemic inflammation. In this review we aim to summarize recent literature concerning the origins, fate and role of microglia, monocytes and neutrophils in models of cerebral ischemia and to discuss their relevance for human stroke.

## Introduction

Cerebral ischemia triggers a robust activation of brain resident and peripheral immune cells, which play an active role in the acute and chronic phases of injury, as well as in subsequent reorganization and repair processes (Iadecola and Anrather, 2011; Macrez et al., 2011; Shichita et al., 2012). Numerous experimental studies have depicted the pivotal response of resident microglia, infiltrating monocyte-derived macrophages and neutrophils to either the development of the brain injury or its resolution leading to conflicting interpretation of their protective or deleterious contribution in stroke (Emerich et al., 2002; Chiba and Umegaki, 2013), and ultimately to treatment failure (O’Collins et al., 2006; Smith et al., 2013). Furthermore, discrepancies exist towards the provenance of brain macrophages, that is to say whether they are blood-borne monocytes or resident microglia that further differentiate into macrophages (London et al., 2013). A better understanding of the origin and function of myeloid immune cells populating the ischemic brain may provide the opportunity to manipulate specific subsets for therapeutic benefit. The aims of this review are (a) to summarize the origin and development of leukocytes and resident microglia, (b) to delineate their contribution to ischemic injury based on recent literature, and (c) to assess their therapeutic relevance to human stroke.

## Origin and development of neutrophils, monocytes and microglia

Cerebral ischemia induces a time-dependent recruitment and activation of leukocytes including neutrophils, monocytes and lymphocytes (Iadecola and Anrather, 2011). At the site of injury, macrophage populations consist mainly of activated parenchymal microglia and infiltrating peripheral monocytes that have distinct ontogenesis (London et al., 2013; Prinz and Priller, 2014). Once in the injured tissue, both cell types differentiate into macrophages and may be indistinguishable by classical histological methods since they share similar antigens and morphologies (Prinz and Mildner, 2011). In the next sections, we will summarize recent findings on the origin, fate and function of myeloid immune cells and microglia particularly in the context of acute cerebral ischemia.

### Origin and fate of neutrophils

**Figure 1:**
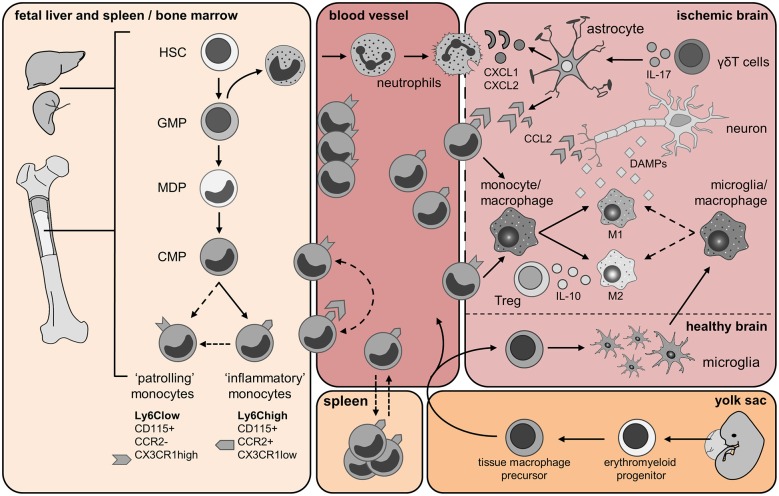
**Origin and trafficking of resident microglia and immune cells of myeloid origin**. Fetal liver and spleen/bone marrow panel: Monocytes are generated from hematopoietic stem cells (HSCs) in the fetal liver and during adult life in the bone marrow (BM). The granulocyte macrophage precursors (GMP) give rise to immature neutrophils and the macrophage dendritic cell precursors (MDPs). Monocytes develop from hematopoietic stem cells (HSC) after myeloid lineage commitment through a series of increasingly restricted progenitors (GMP, granulocyte/monocyte progenitor; MDP, monocyte/dendritic cell progenitors). The common monocyte progenitor (CMP) is the direct precursors of mature Ly6C^high^ and Ly6C^low^ monocytes. Ly6C^high^ inflammatory monocytes might give rise to circulating Ly6C^low^ monocytes directly, or via a Ly6C^high^ monocyte intermediate. Yolk sac panel: Resident brain microglia have been shown recently to have a different origin than circulating monocytes. During embryonic life, erythroblasts (not shown) and macrophage progenitors are generated in the yolk sac from the common erythro-myeloid progenitor. When the blood circulation is established, macrophage precursors exit the yolk sac and migrate into the developing brain. Embryonic microglia proliferate and are able to renew themselves during gestation and post-natal development as well as in adulthood. Blood vessel and Ischemic brain panels: Few hours after cerebral ischemia onset, mature neutrophils enter the bloodstream upon activation of the CXCR2 receptor and infiltrate the brain in response to chemokines CKLF1 as well as CXCL1 and CXCL2 released by astrocytes. Astrocytic production of these chemokines is dependent upon IL-17 released from brain infiltrating γδT cells. Neutrophils firmly adhere to the endothelium and might either invade the ischemic region or cluster into the perivascular space. During injury, CCL2 is produced by astrocytes, macrophages/microglia and neurons. CCL2 binds its receptor CCR2 expressed by Ly6C^high^ inflammatory monocytes, which promotes their egress from the BM into the blood, and then their recruitment from the blood into the injured tissue. Here, these cells give rise to monocyte-derived DC (not shown) and monocyte-derived macrophage populations, which can further polarize into M1 and M2 macrophages. As the ischemic infarct develops, M1 and M2 macrophages contribute to the exacerbation of the damage or wound healing, respectively. Ly6C^low^ monocytes patrol the blood vessel lumen by associating with the vascular endothelium. Ly6C^low^ monocytes expressing the CX3CR1 receptor are also recruited to sites of inflammation and possibly contribute to wound healing by differentiating into alternatively activated M2 macrophages. Cerebral ischemia/reperfusion leads to the release of damage-associated molecular pattern (DAMP) molecules from dying neurons. These molecules trigger the activation of resident microglia and astrocytes. Activated microglia promote tissue repair by producing trophic factors and by scavenging necrotic cells. Regulatory T lymphocytes (Treg) secreting IL-10 have shown a protective role in cerebral ischemia and might promote macrophage M2 polarization. Spleen panel: An interesting twist to the origin of recruited monocytes during injury has been added by the recent identification of a major monocyte reservoir in the spleen of mice. Following ischemic myocardial injury, splenic monocytes are mobilized to the site of inflammation and participate in tissue injury (Swirski et al., 2009). Dashed arrows represent findings that are not clearly defined yet and need further investigations in context of cerebral ischemia.

Neutrophils are innate immune cells and are the first line of defense against microbial infectious agents. They are involved in the phagocytosis, killing and degrading of microorganisms, partly through the generation of reactive oxygen and nitrogen species (ROS/RNS). Neutrophils are generated in the bone marrow (BM) and share with monocytes the common progenitor granulocyte macrophage precursor (GMP; Figure 1). Only mature neutrophils are normally released from the BM into circulation, where they exist as circulating and marginated neutrophil pools, primarily in the lung, which can be acutely mobilized for example by adrenergic agonists (Bierman et al., 1951, 1952). After terminal differentiation, under homeostatic conditions, neutrophils remain in the BM for additional 4–6 days before being released into circulation, constituting a BM neutrophil reserve (Craddock et al., 1960). Egress is controlled by the antagonistic activities of C-X-C chemokine receptor type 2 (CXCR2) and CXCR4 receptors. Activation of CXCR2, which binds C-X-C ligand 1 (CXCL1) and CXCL2 chemokines, stimulates BM egress, whereas stromal cell derived CXCL12 (SDF-1) acting on CXCR4 favors retention (Strydom and Rankin, 2013). During neutrophil maturation CXCR2 expression increases, while CXCR4 is downregulated, which eventually leads to neutrophils egress from the BM. Once in the circulation, neutrophils are thought to be one of the most short-lived cells in the body with circulating half-life of roughly 8 h in humans and 13 h in mice (von Vietinghoff and Ley, 2008; Bugl et al., 2012). As the neutrophil age, CXCR4 receptors become upregulated favoring homing to spleen, liver and BM, wherein neutrophils die by apoptosis and are cleared by resident macrophages. In inflammation, extravasated neutrophils show increased life span of several days and might die as a result of their own cytotoxic molecules by necrosis and by releasing cytotoxic neutrophil extracellular traps (NETs; Yipp et al., 2012).

### Monocytes, the blood-borne precursors of induced tissue macrophages

Similar to neutrophils, monocytes are generated from definitive hematopoietic stem cells (HSC) in the liver and spleen during embryonic development and primarily in the BM after birth (Auffray et al., 2009; Ginhoux and Jung, 2014; Figure 1). After lineage commitment they proceed through increasingly more restricted progenitor stages to give rise to mature monocytes that are released into circulation after engagement of the C-C chemokine receptor type 2 (CCR2; Tsou et al., 2007; Hettinger et al., 2013). In the circulation, they can be distinguished from other leukocytes by their myeloid nature, as indicated by (a) high-level expression of CD11b/Mac-1 (a member of the α-integrin family of proteins) and CD115 (colony simulating factor 1 receptor, CSF1R), (b) their phagocytic capacity and (c) their ability to develop into macrophages upon stimulation with CSF-1 *in vitro* (Chitu and Stanley, 2006). There is substantial heterogeneity in circulating monocytes and they can be further divided into functionally distinct subsets according to their surface expression of lymphocyte antigen 6 complex, locus C1 (Ly6C; Swirski et al., 2007; Geissmann et al., 2008), the CCR2 receptor (Geissmann et al., 2003; Prinz and Priller, 2010) and CX3CR1—the high-affinity functional chemokine receptor for fractalkine (Imai et al., 1997). CD115^+^/Ly6C^high^ monocytes that express CCR2 are termed “inflammatory” monocytes because they are highly mobile and selectively recruited to inflamed tissues (Auffray et al., 2007; Swirski et al., 2010; Terry et al., 2012). In contrast, CD115^+^/Ly6C^low^ monocytes that express CX3CR1 and are negative for CCR2 were initially termed “resident” monocytes because of their longer half-life in circulation and their accumulation in tissues under homeostatic conditions (Geissmann et al., 2003). This subset has been later termed “patrolling” monocytes because of their crawling behavior along the vascular endothelium, and has been shown to orchestrate the disposal of dying or infected endothelial cells (Auffray et al., 2007; Carlin et al., 2013). Their accumulation in the tissue is facilitated by the expression of CX3CR1 that mediates their adhesion and migration (Geissmann et al., 2003). Several reports have proposed that “patrolling” monocytes originate from CD115^+^/Ly6C^high^ monocytes in the circulation (Sunderkötter et al., 2004; Lin et al., 2009). However, recent data suggest that Ly6C^low^ monocytes are generated from a myeloid progenitor by the activity of the transcription factor NR4A1 (Nur77) in the BM (Hanna et al., 2011). Additionally, depletion of CD115^+^/Ly6C^high^ monocytes in the blood does not affect the numbers of circulating CD115^+^/Ly6C^low^ monocytes making it unlikely that CD115^+^/Ly6C^high^ cells are the only precursors of “patrolling” monocytes (Geissmann et al., 2008; Figure 1).

Under physiological conditions, most CD115^+^/Ly6C^low^ crawling monocytes are found within the blood vessels and extravasation rarely occurs. However, in response to tissue damage or infection, these cells extravasate faster than CD115^+^/Ly6C^high^ monocytes and are the main TNF producers in the early phase of the inflammatory response (Auffray et al., 2007). In contrast, in a model of myocardial infarction Ly6C^high^ monocytes are the first population to be present in the injured myocardium and display proteolytic and inflammatory functions consistent with a role in tissue damage. Ly6C^low^ monocytes are the predominant population during the resolution phase of the inflammatory response and express repair-promoting proteins such as vascular endothelial growth factor (VEGF; Nahrendorf et al., 2007). The accumulation of Ly6C^high^ and Ly6C^low^ monocytes in inflamed tissues occurs in two phases, although the sequence of infiltration by specific monocyte subsets differs between models, e.g., bacterial infection vs. sterile inflammation (Auffray et al., 2007; Nahrendorf et al., 2007). Thus, Ly6C^high^ and Ly6C^low^ monocytes can have pro- or anti-inflammatory functions depending on the nature of the inflammatory stimulus, the tissue microenvironment and their molecular profile (transcription factors, gene expression, etc.).

### Microglia, the resident brain macrophages

In contrast to monocytes, microglia originate exclusively from the yolk sac and colonize the central nervous system (CNS) early during embryonic development before E9 (Ginhoux et al., 2010, 2013; Schulz et al., 2012; Frame et al., 2013). There, yolk sac-derived macrophages proliferate and give rise to mature microglia (Figure 1). It seems that microglia are long-lived under physiological conditions and are not normally replaced by BM-derived cells (Alliot et al., 1999; Ajami et al., 2007; Kierdorf et al., 2013a). Recent evidence indicates that microglia homeostasis in adult mice is highly dependent on continuous pro-survival signals provided by the CSF-1 receptor CD115 (Elmore et al., 2014). Blockage of the receptor by selective inhibitors for as little as 7 days resulted in nearly complete depletion of brain microglia. Fast recovery of the microglia population was observed after withdraw of the blocker due to proliferation of brain resident progenitors. These progenitors expressed the neuroectodermal marker Nestin, the hematopoietic marker CD45, the stem cell marker CD34 and stained positive for isolectin B4 (IB4) while being negative for myeloid markers such as ionized calcium binding adapter molecule (Iba1; Elmore et al., 2014). While this is the first study to identify brain-resident microglial progenitors, microglia expansion has been previously described in several disease models. In a model of amyotrophic lateral sclerosis, Solomon et al. characterized the temporal and spatial infiltration of green fluorescent protein (GFP)-BM cells transplanted into lethally irradiated mice. The increased number of spinal cord macrophages following disease onset was attributed to the expansion of resident microglia rather than infiltrated BM-derived precursors (Solomon et al., 2006). Similarly, in experimental autoimmune encephalitis (EAE), Ajami et al. found that blood-borne monocytes transiently infiltrate the spinal cord but do not contribute to the long-term pool of resident microglia (Ajami et al., 2011). Techniques such as parabiosis (Ajami et al., 2007; Ransohoff, 2011), whole body irradiation, head-shielded BM chimeras (Mildner et al., 2011), transgenic mice (Saederup et al., 2010) and fate mapping analysis (Ginhoux et al., 2010) have been instrumental to better discriminate between infiltrating myeloid immune cells and resident microglia. Furthermore, recent data indicate that microglia express a distinctive genetic signature that makes them distinguishable from neurons, astrocytes, oligodendrocytes, and peripheral immune cells including other tissue macrophages. Using transcriptomic and proteomic analysis Butovsky et al. identified several genes that were specifically expressed in CD11b^+^/CD45^low^ microglia when compared to blood monocytes (Butovsky et al., 2014). Among them, the purinergic receptor P2ry12, the proto-oncogene tyrosine kinase Mertk, and the Fc receptor-like S scavenger receptor Fcrls were exclusively expressed in microglia in brain and spinal cord, and not in peripheral tissue macrophages, e.g., Kupfer cells, alveolar macrophages, etc. P2ry12, Mertk and Fcrls are surface expressed proteins enabling easy antibody-mediated detection in flow cytometric and histological assays. Given that this genetic signature is similar between mice and human microglia, a better differentiation of infiltrating myeloid cells and microglia in human stroke should also be possible. In a model of EAE, expression of these genes was restricted to microglia and was not found in infiltrated monocytes (Butovsky et al., 2014). However, because the analysis was conducted at disease onset, it is not clear whether the signature is retained as the pathology progresses. It remains to be determined whether expression of these marker genes is preserved during all stages of microglial activation, as observed in cerebral ischemia, or whether a similar molecular signature can be acquired by infiltrating monocytes that develop into tissue macrophages. For example, monocytes entering the brain parenchyma could be exposed to a brain-specific environment favoring expression of a microglia-like phenotype. Therefore, a better knowledge of unique genetic signatures and reliable cellular markers would greatly facilitate studies of the origin, function and fate of inflammatory cells in ischemic brain injury.

### Polarization of monocytes- and microglia-derived macrophages

Once in the damaged tissue, CD115^+^ peripheral-blood monocytes have the ability to differentiate into macrophages and further polarize into several subtypes with specific functions including production of inflammatory molecules and phagocytic activity. Macrophage polarization depends on the type of injury, the nature of the pathogen, the organs involved and interactions with other immune cells. The distinct phenotypes and physiological activities associated with tissue macrophages were mostly described *in vitro* and in non-neuronal tissue, and have only recently been examined in the context of cerebral ischemia. “Classically activated macrophages” or “M1 macrophages” have anti-microbial activity and secrete pro-inflammatory cytokines and reactive oxygen species upon stimulation with interferon-γ (IFNγ) and lipopolysaccharide (LPS; Mills et al., 2000; Gordon and Taylor, 2005; Mosser and Edwards, 2008). On the other hand, “alternative” or M2 macrophages, induced by interleukin-4 (IL-4), IL-10 or transforming growth factor-β (TGF-β), promote anti-inflammatory and reparative processes (Mantovani et al., 2004; Hao et al., 2012; Yang et al., 2014). Several differentially regulated genes have been associated with each polarization state and are used to distinguish between these two populations in mice, including the IgG Fc receptors CD16/32 and inducible nitric oxide synthase (iNOS) for the M1 phenotype, and mannose receptor (CD206), arginase 1 (Arg1), chitinase-like 3 (Chil3 or Ym1), and IL-10 for the M2 phenotype (Table 1; Hu et al., 2012; Liu et al., 2013; Murray et al., 2014; Tang et al., 2014).

**Table 1:** **Different activation states of neutrophils, circulating monocytes and tissue macrophages in mice**.

	Neutrophils	Circulating monocytes		Monocyte-derived macrophages		Resident microglia	
**Subsets**	N1/N2	“Inflammatory” monocytes	“Patrolling” monocytes	M1	M2 (M2a,b,c)	Surveying microglia	Microglia-derived macrophages
**Marker genes**	iNOS			iNOS	Arg1, CD206, Chil3 (Ym1)	DAP12	
**Receptor expression**	CD45^high^ CD11b^+^ Ly6G^+^	CD45^high^ Ly6C^high^ CD115^+^ CCR2^+^ CX3CR1^int^ Ly6G-	CD45^high^ Ly6C^low^ CD115^+^ CCR2^−^ CX3CR1^+^ Ly6G-	CD45^high^ CD115^+^ CD11b^+^ F4/80^+^ CD68 (ED-1) CCR2^+^	CD45^high^ CD115^+^ CD11b^+^ F4/80^+^ CD68 (ED-1) CX3CR1^+^	CD45^int^ CD11b^+^ CX3CR1 Ibal^+^ IB4	CD45^high^ CD11b^+^ CD115^+^ F4/80^+^ CD68 (ED-1) CX3CR1^+^ Iba1^+^ IB4
**Activated by [cells]**	Platelets, Endothelial cells	Neutrophils, Dying neurons		Th1, NK	Th2	Neurons	Dying neurons
**Activated by [molecules]**	IL-1α	CCL2		LPS, IFNγ, TNF	IL- 4 and IL-13 (M2a), IL-10, Glucocorticoid, TGFβ (M2c), CCL2 (MCP1)		DAMPs, CX3CL1
**Function**	Neurotoxic (N1), Neuroprotection (N2)	Recruited at sites of inflammation, Precursors of peripheral mononuclear phagocytes	Endothelium integrity	Cytotoxic effect	Phagocytic capacity, Promote neurite outgrowth	Synaptic pruning and remodeling during development, Homeostatic functions	Phagocytosis, Neurotoxicity
**Cell products**	ROS, NO, RNS, MMP-9, NE	TNF, ROS, NO, IL-1β, Little IL-10	IL-10, VEGF	IL-12, IL-23, TNF, IL-1β, IL-6, ROS	TGFβ, Arg1 and scavenger receptors (M2a), IL-10 (M2c).	TGFβ	IL-1β, TNF, IL-6, Chemokines, IFNγ

*The dichotomy in the function of each cell type shown here is an oversimplified view of their activation state. They rather undergo multiple activation phenotypes at a given time making the interpretation of their neuroprotective or/and deleterious effect in cerebral ischemia complex. Whether N1/N2 and M1/M2 phenotypes may apply to neutrophils and microglia, respectively, would need further investigation*.

*Abbreviations: Arginase 1 (Arg1), Chemokine (C-C motif) ligand 2 (CCL2), C-C chemokine receptor type 2 (CCR2), Chitinase-like 3 (Chil3 or Ym1), CX3C chemokine receptor 1 (CX3CR1), Damage-associated molecular pattern molecules (DAMPs), DNAX activation protein of 12 kDa (DAP12), inducible nitric oxide synthase (iNOS), Interferon gamma (IFNγ), Interleukin-1,4,6,10,12,23 (IL-1, IL-4, IL-6, IL-10, IL-12, IL-23), Ionized calcium binding adaptor molecule 1 (Iba1), Isolectin B4 (IB4), Lipopolysaccharide (LPS), Lymphocyte antigen 6G (Ly6G), Matrix metallopeptidase 9 (MMP-9), monocyte chemotactic protein 1 (MCP1), Natural Killer (NK), Neutrophil elastase (NE), Nitric oxide (NO), Reactive nitrogen species (RNS), Reactive oxygen species (ROS), Transforming growth factor beta (TGF-β), Tumor necrosis factor (TNF), Type 1 T helper (Th1), Type 2 T helper (Th2), Vascular Endothelial Growth Factor (VEGF)*.

It must be noted that M1 and M2 are the extreme phenotypes of the broad and heterogeneous activation states of macrophages, and caution is needed in interpreting their multivalent function under pathological conditions (Murray et al., 2014). Recently, the M1/M2 terminology was also applied to activated microglia (Michelucci et al., 2009; David and Kroner, 2011). Exposure of cultured primary microglia to LPS or IFNγ induces a M1-like phenotype with reduced phagocytic activity and expression of iNOS, TNF, IL-1β and IL-6 among others, while IL-4 drives microglia towards a M2-like phenotype with increased Arg1, Ym1 and resistin like α (RELMα, FIZZ1) expression (Michelucci et al., 2009). Others have compared capacity of microglia and blood- or BM-derived macrophages to assume M1 or M2 phenotypes using *in vitro* polarization protocols (Durafourt et al., 2012; Girard et al., 2013). These authors assessed morphology, surface markers, cytokine profile and phagocytic capacity of both polarized blood-borne macrophages and microglia (Durafourt et al., 2012) or compared expression levels of M1/M2 marker genes (Girard et al., 2013). Girard et al. found that both mouse BM-derived macrophages and BV2 microglial cell line displayed a M1 or M2 phenotypes after LPS or IL-4 stimulation, respectively. In contrast, Durafourt et al. found that human microglia was able to respond to both M1 and M2-inducing stimuli, but their M2 gene expression signature was restricted to CD209 and the expression of CD23, CD163, and CD206 was not increased, as observed in M2 polarized macrophages (Durafourt et al., 2012). Although the functional implications remain to be established, these observations highlight important differences between human and mouse microglia. Interestingly, adult human microglia showed higher phagocytic activity and IL-10 expression than macrophages under M1 and M2 conditions, indicating that even in a pro-inflammatory environment microglia can retain anti-inflammatory function, which might be essential for their neuroprotective activity (Durafourt et al., 2012).

## Contribution of immune cells of myeloid origin and microglia to cerebral ischemic injury

**Figure 2:**
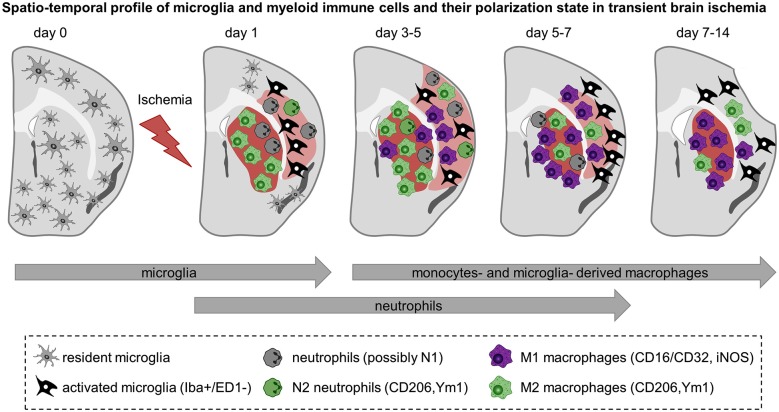
**Spatiotemporal profile of myeloid immune cell activation in acute cerebral ischemia**. Schematic representation of rodent brain coronal sections through the anterior commissure (bregma = 0) after transient MCA occlusion in C57BL/6 mice. The core of the infarct is represented in red and the peri-infarct area is shown in light red. The evolution of the infarct is based on observations of ours and others and depicted as follow: infarct size becomes detectable 1 day after transient MCA occlusion, is maximal at 3–5 days after ischemia onset and decreases afterwards together with shrinkage of the injured hemisphere and enlargement of the ventricles. Before injury, resident microglia display ramified thin processes and are highly motile. They scan their microenvironment to detect any disturbances. Cerebral ischemia triggers microglia to become activated and to display distinct shapes and expression patterns upon the course of reperfusion. Based on findings depicted here, microglia become activated at the onset of cerebral ischemia—as early as 1 day after injury—and further develop into macrophages. In the core of the infarct, microglia are round-shaped, expressed ED-1 and display a M2 phenotype aimed at phagocytize the cellular debris and clear the damaged tissue. In the peri-infarct region microglia have ramified processes but are negative for the phagocytic marker ED-1. They are present early after injury, peak between 5–7 days and decrease thereafter. Neutrophils are the first myeloid immune cells to invade the brain and might display a N1 or N2 phenotypes. They can be detected in the ischemic region from 1 to 5–7 days after injury. Monocytes infiltrate the ischemic parenchyma after microglia activation and transform into macrophages. Between 3–5 days M2 macrophages are more abundant in the striatum—the core of the infarct—and decline by 2 weeks. In contrast, pro-inflammatory M1 cells are first observed in the peri-infarct of the lesion and increase in number in the core over time and outnumber the M2 macrophages later on. From 3–5 days onwards it is not clear yet whether the so called M1 and M2 macrophages found in the ischemic region are derived preferentially from microglia or infiltrated monocytes and if they have similar functions. Preliminary data suggest that microglia-derived macrophages can proliferate and have greater phagocytic properties than monocytes-derived macrophages.

Acute cerebral ischemia leads to rapid neuronal cell death and activation of resident microglia as well as the infiltration of blood-borne immune cells (Gelderblom et al., 2009; Dénes et al., 2010; Figure 2). Activated microglia become phagocytic aiming to clear the damage and promote repair (Faustino et al., 2011). If blood flow is not rapidly restored and ischemic damage develops, injured neurons release damage-associated molecular pattern proteins leading to the secretion of pro-inflammatory mediators and formation of ROS from parenchymal cells (del Zoppo et al., 2000; Shichita et al., 2012; Jackman and Iadecola, 2014). Cytokines and ROS, produced both in the vascular and parenchymal compartments, induce the disruption of the blood brain barrier (BBB) facilitating the infiltration of circulating monocytes, neutrophils and lymphocytes, thereby promoting post-ischemic inflammation (Iadecola and Anrather, 2011; Kleinschnitz et al., 2013; García-Bonilla et al., 2014b). However, neutrophils, as well as monocytes, some T- and B-cells and microglia may also exhibit anti-inflammatory properties that are important for limiting neuronal injury, resolving the inflammation and promoting tissue repair (Liesz et al., 2009; Yilmaz and Granger, 2010; Bodhankar et al., 2014). Characterizing the dynamics of leukocyte infiltration after cerebral ischemia is important to understand their functional role and therapeutic potential. Notably, the magnitude and temporal profile of peripheral immune cell infiltration depends upon models of focal cerebral ischemia. For example, permanent ischemia leads to immune cell infiltration as early as 3 h and a more pronounced accumulation of neutrophils compared to transient ischemia (Zhou et al., 2013; Chu et al., 2014b), wherein blood-borne immune cells do not appear in large numbers until 48 h after reperfusion (Stevens et al., 2002; Gelderblom et al., 2009; García-Bonilla et al., 2014b). These differences in the timing of cellular infiltration relative to the development of tissue injury hint at different roles of blood-borne immune cells in these two stroke models. These considerations need to be taken into account when extrapolating findings obtained in animal models to human stroke for therapeutic purposes.

### Neutrophils in brain ischemia

Neutrophils respond to sterile inflammation, such that induced by brain ischemia, by interacting with endothelial adhesion molecules, mainly selectin family members and intracellular adhesion molecule-1 (ICAM-1), to slow their intravascular movement and induce polarization, which results in firm adhesion to the pro-inflammatory endothelium (Panés et al., 1995, 1999). Rolling and adhesion of leukocytes can be observed *in vivo* as early as 1 h after transient middle cerebral artery (MCA) occlusion and is characterized by neutrophil attachment to the endothelium and increased platelet-neutrophil interactions which is P-selectin and integrin αIIbβ3 dependent (Ishikawa et al., 2004). Due to the proximity of platelets and neutrophils, IL-1α released from activated platelets, might be an early activator of neutrophils (Thornton et al., 2010).

After initial adherence, neutrophils will follow a chemokine and activator gradient produced by the injured tissue. Neutrophils are the first blood-borne cells found in the ischemic area, reach peak numbers at days 2–4 after transient ischemia and decline thereafter (Gelderblom et al., 2009; García-Bonilla et al., 2014b). The CXCR2 ligands CXCL1 (KC) and CXCL2 (MIP-2α) are the main chemokines responsible for neutrophil extravasation. In the ischemic brain CXCL1 is produced by activated astrocytes in an IL-17 dependent manner (Figure 1; Gelderblom et al., 2012). Chemokine-like factor 1 (CKLF1), a recently discovered chemokine, participates in neutrophil recruitment after transient focal ischemia in rats and anti-CKLF1 antibodies diminished neuronal cell death in this model (Kong et al., 2014). The sequence of molecular events regulating neutrophil extravasation remains incompletely understood. CD47, a immunoglobulin superfamily transmembrane glycoprotein expressed on the cell surface of neutrophils, is essential for neutrophil transmigration *in vivo* and *in vitro* (Liu et al., 2001; Su et al., 2008). Accordingly, CD47 deficient mice exhibit reduced brain injury and neutrophil infiltration after transient focal ischemia (Jin et al., 2009).

Endothelial transmigration seems to be essential for the cytotoxic activation of neutrophils (Allen et al., 2012), which is accomplished by induction of ROS production by NADPH oxidase and myeloperoxidase, iNOS dependent nitric oxide generation and concomitant release of RNS, particularly peroxynitrite (Yilmaz and Granger, 2010). Work from our group has shown that iNOS expression in endothelial cells and neutrophils contributes to ischemic brain injury after transient MCA occlusion, but was dispensable for neutrophil recruitment (Iadecola et al., 1997; García-Bonilla et al., 2014a). On the other hand, adoptive transfer of iNOS^+/+^ neutrophils into an iNOS^−/−^ host 24 h after MCA occlusion was sufficient to revert the protection exerted by iNOS deletion (García-Bonilla et al., 2014a). Other potentially neurotoxic molecules released by neutrophils are extracellular proteases. Among them, matrix metalloproteinase 9 (MMP-9), a protease released during neutrophil degranulation, has been shown to contribute to ischemic injury (Romanic et al., 1998). Neutrophil elastase (NE), has also been implicated in brain injury after transient but not permanent focal ischemia (Shimakura et al., 2000; Stowe et al., 2009). There is still debate to what extent neutrophils contribute to post-ischemic inflammation and neuronal cell death. While neutrophil depletion by delivery of anti-neutrophil antibodies was neuroprotective in focal ischemia models (Matsuo et al., 1994), several approaches to limit neutrophil infiltration by cytotoxic drugs or adhesion molecule and chemokine receptor blockade have been unsuccessful (Soriano et al., 1999; Beray-Berthat et al., 2003b; Brait et al., 2011), or have suggested that the contribution of neutrophils to ischemic injury might be brain region specific (Beray-Berthat et al., 2003a). It has also been suggested that neutrophils do not penetrate the ischemic brain tissue but accumulate in the perivascular space after extravasation (Enzmann et al., 2013). However, this finding does not exclude their participation in ischemic injury since neutrophils would be still able release soluble cytotoxic molecules such as ROS, RNS, and proteases, which could induce vascular damage aggravating the ischemia or diffuse into the brain parenchyma causing tissue injury. Indeed, our iNOS BM chimera and adoptive transfer experiments (García-Bonilla et al., 2014b), collectively, support a deleterious role of neutrophils in ischemic brain injury.

### Functional polarization of neutrophils in cerebral ischemia

In addition to their deleterious effect, neutrophils may also exert neuroprotective functions as recently suggested (Cuartero et al., 2013). In this study it was shown that in a mouse model of permanent focal ischemia the neuroprotective effect of the PPARγ agonist rosiglitazone was abolished in neutrophil depleted animals. Rosiglitazone treatment induced an increase of total number of neutrophils 24 h after ischemia and a shift in the ratio of pro-inflammatory N1 neutrophils to N2 neutrophils shown by increased expression of the M2-marker protein Ym1 (*Chil3*). N2 neutrophils were recently defined as a population of polarized cells with anti-inflammatory phenotype, in accordance to the M1/M2 classification of macrophages, and have been found in tumors where they facilitate tumor growth by inhibiting anti-tumor T cell responses (Fridlender et al., 2009; Mantovani, 2009). Their role in brain ischemia, including the mechanism of neuroprotection, remains to be firmly established, but, analogous to the double-edged role of monocytes/macrophages, neutrophils could also have beneficial effects, perhaps in the repair phase of the injury. For example, neutrophils that undergo apoptosis are ingested by microglia and macrophages, which induce these phagocytic cells to become anti-inflammatory or promote tissue repair (Serhan and Savill, 2005). Therefore, manipulating the polarization state of neutrophils could also have therapeutic relevance.

### Monocytes and microglia in cerebral ischemia

Under normal conditions, resident microglia are ramified, have long processes and can be morphologically discerned from macrophages (Nimmerjahn et al., 2005; Boche et al., 2013). Resident microglia can be distinguished from BM-derived monocytes according to their level of CD45 expression: microglia are CD11b^+^ and express intermediate level of CD45 (CD11b^+^/CD45^int^), whereas monocytes are also positive for CD11b but express high level of CD45 (CD11b^+^/CD45^high^) (Ford et al., 1995). Monocytes can be further classified as Ly6C^high^ pro-inflammatory monocytes or Ly6C^low^ anti-inflammatory monocytes (David and Kroner, 2011). Upon injury, microglia adopt ameboid morphology and become phagocytic. Activated microglia are commonly identified by the cellular markers: IB4, Iba1, F4/80 or ED-1 (CD68). After cerebral ischemia, microglia display different markers and morphology depending on their localization in the ischemic territory and the course of reperfusion. In the ischemic core microglia have an ameboid shape, are Iba1 positive and ED1 positive and have increased CD11b expression, whereas in the peri-infarct area these cells have shorter processes than in the resting state, are Iba1^+^, but are negative for ED1 (Ito et al., 2001; Morrison and Filosa, 2013). Once microglia differentiate into macrophages they share several antigens and morphological features with hematogenous macrophages (Patel et al., 2013), confounding the interpretation of their origin and ultimately their function in acute cerebral ischemia (Hanisch and Kettenmann, 2007; Hellwig et al., 2013).

Several recent studies have attempted to distinguish microglia from blood-derived macrophages and to define their role in ischemic injury and repair, potentially opening novel therapeutic avenues for the treatment of stroke. To this end, several strategies have been used (Prinz and Priller, 2014). A widely used approach has been to perform BM transplants with fluorescent BM (Tanaka et al., 2003). One drawback to this approach is that whole body irradiation may affect the integrity of the BBB and induce brain cytokine production, creating a permissive environment for brain engraftment of BM-derived immune cells normally not found in the CNS (Mildner et al., 2007). Although head shielding during irradiation or parabiosis may avoid such disturbances, the resulting degree of chimerism (40–50%) is not as high as with whole body irradiation (>90%) (Ginhoux et al., 2013; Prinz and Priller, 2014). The reduced chimerism obtained with these models may not be problematic for cell tracking and fate determination experiments, but it will confound the interpretation of mechanistic studies on the relative contribution of resident and blood-borne immune cells to tissue damage and repair. Recently, it has been shown that chemical BM ablation with busulfan leads to a better rate of blood chimerism with reduced inflammation and myeloid immune cell recruitment in the mouse CNS compared to irradiation (Kierdorf et al., 2013b). However, in our own experience, doses of busulfan that produce a chimerism comparable to irradiation lead to a myeloid immune cell recruitment in brain greater than that seen with irradiation (Sugiyama et al., 2014). Therefore, chemical BM ablation-based approaches require further scrutiny.

Another approach to track blood-borne myeloid cell infiltration into the ischemic area, has been to use ultrasmall superparamagnetic iron oxide particles (USPIO), which labels peripheral phagocytic cells after intravenous injection. USPIO were delivered in one case 5.5 h after permanent MCA occlusion (Rausch et al., 2001) and in an other study 24 h before inducing photothrombotic strokes (Kleinschnitz et al., 2003). Brains were analyzed at different time points after ischemia by magnetic resonance imaging (MRI) and histology. While the temporal profile was slightly different between the two studies, iron particles were detected late in the post-ischemic period, after most of the damage had occurred (Rausch et al., 2001; Kleinschnitz et al., 2003). In a model of neonatal ischemia, microglia—identified as CD11b^+^/CD45^low/int^ by flow cytometry—were the predominant cell population in the infarct area compared to CD11b^+^/CD45^high^ monocytes (Denker et al., 2007). A similar pattern was described using GFP-BM chimeric mice. Thus, Schilling et al. found that microglia were the first cells to be activated in the infarcted area, exhibited a more pronounced phagocytic activity and out-numbered GFP-BM monocytes (Schilling et al., 2003, 2005). Collectively, these observations suggest that microglia are likely to be the first cells activated after brain injury, aiming at clearing the cell debris by phagocytosis and contributing to the resolution of inflammation (Neumann et al., 2009). On the other hand, phagocytosis could also contribute to neuronal cell loss after ischemia. For example, it has been suggested that microglia could engulf viable neurons in the ischemic penumbra (Neher et al., 2013). Salvageable neurons would send “eat me” signals to nearby microglia and induce their phagocytosis that further increases brain damage (Neher et al., 2012). In support of this hypothesis, deficiency of the phagocytosis-related receptors Mertk and milk fat globule-EGF factor 8 protein (Mfge8) on microglia promotes functional recovery and reduces brain atrophy in focal infarcts (Neher et al., 2013). On the other hand, deletion of triggering receptor expressed on myeloid cells 2 (TREM2), a receptor expressed on phagocytic microglia and macrophages, did not affect infarct size in a focal ischemia model in mice (Sieber et al., 2013), although TREM2 is essential for their phagocytosis capacity (Takahashi et al., 2007). The molecular mechanisms regulating these microglial behaviors need to be established and may provide new insights into endogenous brain processes regulating injury and repair.

### CCR2^+^ monocytes in cerebral ischemia

Recruitment of circulating monocytes to the ischemic brain is orchestrated by inflammatory cytokines, de novo-expressed adhesion molecules and chemokines. The monocyte chemoattractant protein (MCP-1, CCL2) and its receptor CCR2 are known to be involved in the inflammatory response of the injured brain after cerebral ischemia (Che et al., 2001; Chu et al., 2014a). After permanent and transient focal ischemia, Gliem et al. found an increase in Ly6C^high^ monocytes at 3 days, whereas the number of Ly6C^low^ monocytes was greatest at 6 days, paralleled by sequential peaks of CCR2 and CX3CR1 mRNA as well as gene expression of the pro- and anti-inflammatory cytokines IL-1β and TGF-β, respectively (Gliem et al., 2012). Furthermore, they demonstrated that selective deletion of CCR2 in BM-derived cells induced a delayed clinical deterioration and hemorrhagic conversion of ischemic infarcts, suggesting a beneficial role of CCR2^+^ monocytes in maintaining the structure of the neurovascular unit (Gliem et al., 2012). Because CCR2^+^/Ly6C^high^ monocytes can transform into anti-inflammatory Ly6C^low^F4/80^high^ macrophages, it was suggested that lack of infiltrating CCR2^+^ monocytes results in reduction of anti-inflammatory macrophages at later time points compromising repair mechanisms. However, several other studies demonstrated reduced phagocytic macrophage accumulation in the brain of CCL2 or CCR2 knock-out mice together with smaller infarcts after cerebral artery occlusion (Hughes et al., 2002; Dimitrijevic et al., 2007; Schilling et al., 2009), suggesting a deleterious role of CCR2 monocytes. These discrepancies may reflect the multifunctional roles of CCR2^+^ monocytes, which, while contributing to early brain injury may also enable delayed repair processes.

### CX3CR1^+^ monocytes and microglia in cerebral ischemia

The chemokine CX3CL1 is found on neurons while its receptor CX3CR1 is expressed on microglia and Ly6C^low^ monocytes (Geissmann et al., 2003; Sunnemark et al., 2005; Limatola and Ransohoff, 2014). CX3CR1^−/−^ or CX3CL1^−/−^ mice show reduced brain damage in transient focal ischemia models compared to wild type mice (Soriano et al., 2002; Dénes et al., 2008; Fumagalli et al., 2013), although the effect might not be sustained in time (Gliem et al., 2012). Neuroprotection found in CX3CR1^−/−^ mice following focal cerebral ischemia was correlated with an increase of the M2 macrophage marker genes CD206 and Ym1 and a decrease of M1-iNOS gene expression compared to wild type mice (Fumagalli et al., 2013; Tang et al., 2014). These findings indicate that CX3CR1 deficient mice induce a different pattern of M1/M2 marker expression in the ischemic region. The authors suggest that the lack of CX3CR1 in microglia and infiltrated monocytes favors an anti-inflammatory milieu that protects the brain from infarct development. However, intracerebroventricular delivery of CX3CL1 at the time of MCA occlusion decreased infarct volumes (Cipriani et al., 2011), suggesting that CX3CL1 acts on microglia to reduce their activation state and inhibit the release of inflammatory cytokines (Zujovic et al., 2000; Cardona et al., 2006; Biber et al., 2007). Michaud et al. has recently shown that selective ablation of Ly6C^low^/CX3CR1^+^ monocytes, thought to preferentially develop into M2 macrophages, does not influence stroke outcome in a hypoxic-ischemic injury model (Michaud et al., 2014). Using NR4A1^−/−^ BM chimeras that lack circulating Ly6C^low^/CX3CR1^+^ monocytes (Carlin et al., 2013), it was found that structural and functional outcome were not affected. However, brain infiltrating monocytes have not been assessed in this study, and it remains an open question whether Ly6C^high^/CCR2^+^ monocytes in the brain parenchyma could give rise to Ly6C^low^/CX3CR1^+^ monocytes/macrophages, even in the absence of the NR4A1 transcription factor.

Using parabiosis of CX3CR1^GFP/+^ mice and wild type partners, Li et al. found that microglia increased gradually during the first week after photothrombotic stroke (Li et al., 2013). Hypertrophic ameboid microglia were more abundant in the periphery compared to the core of the lesion and many GFP-positive microglia were labeled with the mitotic marker bromodeoxyuridine (Li et al., 2013). In the wild type parabiotic partner, CX3CR1^GFP/+^ infiltrating cells started to populate the brain 5 days after ischemia onset, were less numerous than activated microglia and did not proliferate, suggesting that microglia and monocyte-derived CX3CR1 macrophages are two distinct populations with presumably different roles in cerebral ischemia.

### M1 and M2 phenotypes in cerebral ischemia

Recent studies have begun to investigate microglia and macrophage polarization *in vivo*. In a model of permanent focal ischemia, different cell markers were used to characterize the prevalence of phagocytic cells and M2-like phenotype at different time after ischemia (Perego et al., 2011). Soon after injury, ED-1 positive phagocytic cells were present at the border—possibly limiting the expansion of the infarct—whereas Ym1- and CD206-expressing cells were found in the ischemic core. At 7 days, more phagocytic cells invaded the ischemic core where they engulfed neurons. In transient focal ischemia, M2-type gene expression (CD206, Arg1, CCL22, Ym1/2, IL-10, and TGF-β) was first apparent from 1 to 3 days after ischemia, peaked at 3–5 days, was reduced at 7 days and returned to pre-injury levels by day 14. M1-type genes (iNOS, CD11b, CD16, CD32, and CD86) were gradually increased from day 3 and remained elevated for 14 days after ischemia (Hu et al., 2012). The M1/M2 phenotype was also assessed in *in vitro* models of ischemic injury using microglia and neuronal co-culture. M1-polarized microglia/macrophages exacerbate neuronal death compared with M2 cells (Hu et al., 2012; Girard et al., 2013). Hu et al. suggested that early expression of M2 genes may contribute to neuroprotection by enhancing the phagocytosis of dead cells, removal of tissue debris and promote recovery. At later stages, cells displaying a M1 phenotype may induce pro-inflammatory mediators and exacerbate neuronal death. Therefore, limiting such M2 to M1 shift and promoting M2 polarization could be a potential therapeutic strategy (Hu et al., 2012). *In vitro* experiments have to be interpreted with caution because microglia are commonly derived from neonatal tissue and develop in a culture environment that cannot recapitulate the maturation process that occurs *in vivo* (Hellwig et al., 2013). Importantly, the gene expression signature characteristic for adult brain microglia is lost in primary neonatal microglia cultures as well as in microglial cell lines including BV2 and N9 cells (Butovsky et al., 2014). Thus, *in vivo* translation of findings obtained with *in vitro* polarization models might not be easily achieved. Desestret et al. administered differentiated BM-derived M2 macrophages 4 days after cerebral ischemia by intravenous injection. M2 macrophages failed to induce protection or improve behavioral outcome (Desestret et al., 2013). On the other hand, Girard et al. have proposed that M1 and M2 BM-derived macrophages promote cell death *in vitro*, whereas M2 microglia is neuroprotective. However, microglia does not display a classic M1 and M2 phenotypes after MCA occlusion (Girard et al., 2013). The ability of microglia to differentiate into multiple macrophage-related phenotypes depending on their microenvironment highlights the complexity of their response to cerebral ischemia (Taylor and Sansing, 2013).

### A role for splenic monocytes in brain ischemia?

The discovery of a splenic monocyte reservoir in adult mice, that is mobilized following ischemic myocardial injury (Swirski et al., 2009), has sparked renewed interest in the role of the spleen in brain ischemia. Previous studies have shown that splenocytes respond to cerebral ischemia by producing large amounts of inflammatory mediators early after injury (Offner et al., 2006b), followed later by an increase of blood monocytes and splenic regulatory T cells, that are thought to dampen the inflammatory response (Offner et al., 2006a). In accordance with these findings, chronic splenectomy (Ajmo et al., 2008) or acute spleen irradiation (Ostrowski et al., 2012) have been performed in rats to inhibit systemic inflammatory responses after stroke. Although the studies differed in the ischemic model, i.e., permanent vs. transient ischemia, both studies observed neuroprotection together with a decrease of immune cell counts in the ischemic region. In addition, post-stroke treatment with the antibacterial agent moxifloxacin (MFX) reduced peripheral infection and infarct size in animal models of stroke (Meisel et al., 2004; Bao et al., 2010). In MFX-treated mice the percentage of the splenic pro-inflammatory Ly6C^high^ monocytes was reduced compared to vehicle animals 7 days after stroke, together with reduced expression of CCR2 in the spleen and the brain (Bao et al., 2010). These studies suggest that interventions aiming at reducing systemic inflammation, specifically by targeting splenocytes, could lead to a reduction of brain immune cell infiltration and neuroprotection. However, these reports did not assess whether leukocytes, specifically monocytes, found in the brain originate from the spleen and contribute directly to stroke outcome. For instance, Ajmo et al. did not find alteration of immune cell counts in peripheral blood following MCA occlusion in sham or splenectomized animals, while spleen irradiation after MCA occlusion resulted in a drop of circulating lymphocytes, but failed to lower blood monocytes and neutrophils (Ajmo et al., 2008; Ostrowski et al., 2012). Thus it is not clear whether peripheral immune cells are mobilized from the spleen into the brain and contribute directly to stroke outcome. Recently, Kim et al. analyzed the temporal profile of brain infiltrating Ly6C^high^ and Ly6C^low^ monocytes after focal ischemic injury in acutely splenectomized mice (Kim et al., 2014). In sham mice, they found that brain ischemia induced a reduction of both monocytes subsets in the spleen that temporally correlated with their increase in the ischemic brain. In splenectomized mice, brain Ly6C^high^ and Ly6C^low^ monocytes were reduced at 1 and 3 days after stroke compared to sham surgery. However no difference in infarct size was observed between groups (Kim et al., 2014). While this study did not directly track splenic monocytes and the findings remain correlative, it suggests that the spleen might contribute both Ly6C^high^ and Ly6C^low^ monocytes after brain ischemia. The failure to observe neuroprotection in this model might be related to simultaneous reduction of both monocyte subsets, thus balancing the loss of deleterious Ly6C^high^ monocytes with the loss of beneficial Ly6C^low^ monocytes, which might be involved in resolution of the inflammation and tissue repair (Shechter et al., 2013).

## Myeloid immune cells in human stroke

### Temporal profile of myeloid immune cell activation and infiltration

Histological analysis of autopsy material from patients allowed to draw a temporal profile of cellular events from 1 day after stroke onset to several years (Chuaqui and Tapia, 1993; Mena et al., 2004). Astrogliosis was observed within the first day of cerebral infarct development together with neutrophils, which were observed in 81% of cases between 3–37 days after stroke onset (Mena et al., 2004). This active inflammatory phase was accompanied by tissue necrosis and infiltration of inflammatory cells such as mononuclear cells and macrophages, which were not observed during the first 2 days after stroke onset. Interestingly, inflammatory mononuclear cells and macrophages persisted up to 53 years and were found in the majority of late stage specimens while neutrophils were absent (Mena et al., 2004). Although the temporal sequence and type of cells in the infarct are similar in rodents and humans (Gelderblom et al., 2009), the chronology of events seems to be more delayed and more spread out in time in humans.

Advanced imaging techniques to visualize post-ischemic inflammation and leukocyte infiltration are increasingly being employed in the clinical setting. Visualization of infiltrated macrophages in stroke patients was performed by USPIO-MRI (Saleh et al., 2004, 2007; Cho et al., 2007). In all patients tested, an increase signal of USPIO was observed by MRI in the brain parenchyma (Saleh et al., 2004). Only a minority of patients that received USPIO between 24 h and 96 h after symptom onset showed a positive signal in the brain (Cho et al., 2007; Saleh et al., 2007). However, iron-positive cells detected in the brain cannot represent only monocytes since activated microglia can engulf iron particles as well (Oude Engberink et al., 2008; Desestret et al., 2009). Injection of iron oxide monocytes labeled *ex vivo* allowed to follow their brain infiltration in experimental models of stroke (see previous section) (Stroh et al., 2006; Oude Engberink et al., 2008). Similarly, the use of *ex vivo* monocyte labeling in humans may increase our understanding of the temporal pattern of blood-borne cell infiltration after stroke. Positron emission tomography (PET) has been used to label inflammatory cells in stroke patients using the radioligands ^11^C(R)-PK11195 for the translocator protein 18kDa (TSPO), commonly used as a microglial marker (Stephenson et al., 1995; Cagnin et al., 2007). Analysis in patients showed an increase of the marker between 3 and 150 days after stroke (Gerhard et al., 2005). Positive labeling was observed early after ischemia, initially at the periphery of the lesion, then in the infarct core and eventually in peri-infarct regions (Gerhard et al., 2005; Thiel and Heiss, 2011). Altogether, the time of magnetic particles or radiolabelled agents infusion might be critical to discriminate resident microglia from immune cells of myeloid origin (Deddens et al., 2012). However, a limitation is that these techniques of brain macrophage visualization do not permit to differentiate between their different subtypes and activation states (Weissleder et al., 2014).

### Monocyte subsets in patients with cerebral ischemia

In human three distinct blood monocytes can be distinguished relative to the expression of the receptor CD14 (LPS co-receptor) and CD16 (Fcγ receptor III). The majority of human “classical” monocytes express high level of CD14 and are negative for CD16 (CD14^++^/CD16^−^), whereas the minor subtypes express CD16 and can be either CD14^high^ (CD14^++^/CD16^+^) or CD14^low^ (CD14^dim^/CD16^+^), also known as intermediate and non-classical monocytes, respectively (Ziegler-Heitbrock, 1996; Ziegler-Heitbrock et al., 2010). The classical monocytes CD14^+^/CD16^−^ share some features with the murine Ly6C^high^ monocytes such as the high expression of the chemokine receptor CCR2 (Weber et al., 2000), whereas CD16^+^ cells express CX3CR1 similar to mouse Ly6C^low^ monocytes (Merino et al., 2011). However, the pro- or anti-inflammatory functions of these different monocyte subsets depend on the inflammatory context and, as in the mouse, a strict association of phenotype and function can not be established (Gordon and Taylor, 2005; Ziegler-Heitbrock, 2007; Merino et al., 2011). For example, blood monocyte subsets from healthy donors showed different pattern of response when stimulated with LPS (Frankenberger et al., 1996; Skrzeczyńska-Moncznik et al., 2008). CD14^dim^/CD16^+^ monocytes were found to produce more TNF compared to CD14^high^/CD16^+^ and CD14^high^/CD16^−^ monocytes, whereas CD14^high^/CD16^+^ were the main source for IL-10, (Belge et al., 2002; Skrzeczyńska-Moncznik et al., 2008).

Similar to the mouse (Offner et al., 2006b), stroke has a significant impact on the peripheral immune system in humans (Kamel and Iadecola, 2012). Although there is significant immunosuppression in acute stroke (Meisel et al., 2005; Chamorro et al., 2006), a significant increase of circulating monocytes has also been reported (Losy and Zaremba, 2001). The different subtypes of blood monocytes were investigated in patients following stroke and were correlated with outcome severity (Urra et al., 2009). The CD14^high^/CD16^+^ subset was increased 48 h after admission, while a parallel decrease in the CD14^dim^/CD16^+^ subtype was observed. An increase in classical CD14^high^/CD16^−^ monocytes was associated with poor outcome, whereas increased CD16^+^ monocytes were linked to a better prognosis (Urra et al., 2009). CD14 expressing cells were observed in brain tissue from patients with focal infarction as early as 1 day and persisted for months after ischemia (Beschorner et al., 2002), suggesting a long-term involvement in inflammatory processes at the site of injury.

There is still a large knowledge gap about human microglia and immune cells of myeloid origin regarding their activation state, spatiotemporal distribution in the brain and, ultimately, about their role in acute cerebral ischemia. Advanced brain imaging modalities in humans and new cerebral ischemic models more representative of human stroke would help enhance our understanding of the inflammatory processes occurring in acute cerebral ischemia and to test therapeutic strategies directed at manipulating their beneficial or deleterious effects.

### Targeting inflammation as therapeutic strategies

The only successful treatment for ischemic stroke is thrombolysis with tissue plasminogen activator (tPA), which, due to the narrow therapeutic window (<4.5 h) (Hacke et al., 2008) and safety concerns, is administered to less than 5% of patients (Fonarow et al., 2011). Different strategies aimed at preventing the inflammatory response after cerebral ischemia have been successful in rodent models. Ischemic damage was shown to be attenuated, among others, by inhibition of microglial activation using the immunomodulatory antibiotic minocycline, systemic T lymphocytes depletion using sphingosine 1-phosphate receptor agonist (FTY720), diminishing free radical generating and pro-inflammatory enzymes, such as iNOS or cyclooxygenase-2, inhibition of cytokines secretion, or targeting adhesion molecules (for reviews, Dirnagl et al., 1999; Iadecola and Alexander, 2001; Wang, 2005; Jordán et al., 2008).

Unfortunately attempts to translate anti-inflammatory therapeutic interventions into the clinics have been more disappointing than promising (O’Collins et al., 2006; Moskowitz et al., 2010). The selective IL-1 receptor antagonist, IL1-ra, which limits the pro-inflammatory action of IL-1, has been tested in randomized patients with acute stroke (Emsley et al., 2005). Despite a conclusive phase II study no recent publications have reported the recombinant human IL1-ra as a therapeutic agent for acute stroke. Strategies to inhibit neutrophil infiltration have been tested in clinical trials. Treatment with the murine mono-clonal anti-ICAM-1 in the Enlimomab trial (Enlimomab Acute Stroke Trial Investigators, 2001) or UK-279,276 (neutrophil inhibitory factor) have failed to improve recovery in acute ischemic stroke patients (Krams et al., 2003). Likewise, NXY-059, a nitrone-based free radical trapping agent, has not demonstrated any benefits in stroke clinical trials (Shuaib et al., 2007). The granulocyte colony-stimulating factor (G-CSF), that stimulates proliferation, survival, and maturation of BM-derived cells, has shown neuroprotective effect in models of transient focal ischemia by inducing mobilization of haemopoietic stem cells, by having anti-apoptotic and anti-inflammatory properties, as well as by promoting neuronal differentiation and angiogenesis (Komine-Kobayashi et al., 2006; England et al., 2009). However, recent results from a large phase IIb trial (AXIS-2) was not able to detect clinical efficacy in acute ischemic stroke patients (Ringelstein et al., 2013).

Reasons for failure have been discussed extensively in the literature and might include ambivalent roles of post-stroke inflammation as well as functional differences between rodent and human immune systems, patient inclusion criteria, timing of intervention, evaluation of efficacy, pharmacokinetic issues and side effects such as promoting infections and fever (for review, Dirnagl, 2006; Endres et al., 2008). Before translating experimental findings into therapies that target the immune system in stroke patients, there is a need to better understand the spatiotemporal profile and functional states of immune cells involved in ischemic brain injury in humans. A better insight in these aspects is crucial for planning future clinical trials targeting post-ischemic inflammation.

## Concluding remarks

The surprising plasticity of myeloid immune cells and microglia in their response to tissue injury highlights the need for further research to define spatiotemporal profiles of their distinct subsets in ischemic brain injury. Given the neuroprotective potential of some of these subsets, it has become clear that inhibiting inflammation in general or indiscriminately blocking leukocyte access to the brain might not be a viable therapeutic approach. The failure of clinical trials that targeted post-ischemic leukocyte recruitment might be in part explained by the indiscriminate nature of these approaches and a lack of a nuanced understanding of the impact of inflammation on damage and repair processes in the post-ischemic brain. Our understanding of the behavior of myeloid immune cells and microglia in ischemic stroke is still limited. Key questions to be addressed in future research include the transcriptomic and proteomic characterization of myeloid immune cell subsets at different stages of brain ischemia, the mechanism of monocytes, neutrophils and microglia polarization in the ischemic environment, and the neural and humoral signals generated by the ischemic brain to modulate peripheral immune cell activation and mobilization. Answering some of these questions would provide the knowledge base needed to design more specific immunotherapies for the treatment of ischemic stroke.

## Conflict of interest statement

The authors declare that the research was conducted in the absence of any commercial or financial relationships that could be construed as a potential conflict of interest.
